# Test combination to detect latent *Leishmania* infection: A prevalence study in a newly endemic area for *L*. *infantum*, northeastern Italy

**DOI:** 10.1371/journal.pntd.0010676

**Published:** 2022-08-15

**Authors:** Alessandra Mistral De Pascali, Renato Todeschini, Simone Baiocchi, Margherita Ortalli, Luciano Attard, Ana Victoria Ibarra-Meneses, Eugenia Carrillo, Stefania Varani

**Affiliations:** 1 Section of Microbiology, Department of Experimental, Diagnostic and Specialty Medicine, Alma Mater Studiorum University of Bologna, Bologna, Italy; 2 Unit of Hygiene and Public Health, Department of Public Health, AUSL Bologna, Bologna, Italy; 3 Microbiology Unit, IRCCS Azienda Ospedaliero-Universitaria di Bologna, Bologna, Italy; 4 Infectious Diseases Unit, IRCCS Azienda Ospedaliero-Universitaria di Bologna, Bologna, Italy; 5 WHO Collaborating Centre for Leishmaniasis, National Centre for Microbiology, Instituto de Salud Carlos III, Majadahonda (Madrid), Spain, CIBERINFEC; 6 Département de pathologie et microbiologie, Faculté de médecine vétérinaire, Université de Montréal, Saint-Hyacinthe, Quebec, Canada; 7 The Research Group on Infectious Diseases in Production Animals (GREMIP), Faculty of Veterinary Medicine, Université de Montréal, Montreal, Canada; University of Iowa, UNITED STATES

## Abstract

**Background:**

Most people infected with *Leishmania* remain asymptomatic, which is a common element that may promote the resurgence of clinically evident leishmaniasis in individuals with impaired cell-mediated immune responses. Unfortunately, there is no universally accepted assay to identify asymptomatic infection. This cross-sectional study focuses on the employment of three methods targeting different features of the parasitic infection to be used in combination for the screening of latent leishmaniasis in a newly endemic area of northeastern Italy.

**Methodology/principal findings:**

The selected methods included highly sensitive Real-Time PCR for detection of parasitic kinetoplast (k)DNA in peripheral blood, Western Blot (WB) for detection of specific IgG, and Whole Blood stimulation Assay (WBA) to evaluate the anti-leishmanial T-cell response by quantifying the production of IL-2 after stimulation of patients’ blood with *Leishmania* specific antigens. Among 145 individuals living in a municipality of the Bologna province, northeastern Italy, recruited and screened for *Leishmania* infection, 23 subjects tested positive (15.9%) to one or more tests. Positive serology was the most common marker of latent leishmaniasis (15/145, 10%), followed by the detection of specific cell-mediated response (12/145, 8%), while only few individuals (6/145, 4%) harbored parasitic DNA in the blood.

**Conclusions/significance:**

Combining different tests substantially increased the yield of positivity in detecting latent *Leishmania* infection. The test combination that we employed in this study appears to be effective to accurately identify latent leishmaniasis in an endemic area.

## Author summary

After infection with *Leishmania infantum* cutaneous or visceral leishmaniasis can develop, but in most cases the host becomes an asymptomatic carrier of the parasite. This key population of individuals with latent leishmaniasis can undergo reactivation of the infection with severe outcome in case of immunosuppression and can contribute to disease transmission.

The public health impact of leishmaniasis is currently underestimated in Europe, despite *Leishmania* infection is endemic in the Mediterranean countries and outbreaks of human leishmaniasis have been reported in the last decades, including northeastern Italy. One of the causes contributing to this neglect is that the available diagnostic tests exhibit scarce sensitivity in identification of latent leishmaniasis.

In this study, we identified latent *Leishmania* infection in a newly endemic area of northeastern Italy by using three different diagnostic methods; serological, molecular and immunological tests. Sixteen percent of individuals residing in the selected area were shown to be positive to at least one of the three tests, which indicates a previous contact with the parasite. This study highlights the importance of test combination to enhance the ability to identify latent *Leishmania* infection.

## Introduction

In the Mediterranean Europe, leishmaniasis caused by *Leishmania infantum* is a neglected health threat, with domestic (dogs and cats) and sylvatic (rabbits and hares) animals playing an important role in the infective cycle [1,2,3]. Italy provided the first European evidence for the northward spreading of leishmaniasis [4,5], including recent outbreaks of visceral leishmaniasis (VL) in northeastern Italy [6,7]. Various factors are likely contributing to the recent emergence of VL in this geographic location, such as climate changes in territories where sandflies circulate, environmental transformation related to human activities, and the growing of the immunocompromised population that potentially act as parasite carriers [1].

When infection occurs, *Leishmania* can be killed by effective immunity, or the parasite can survive due to several evasion mechanisms [8]. In the latter case, the relationship between the host and the parasite can be balanced, the human host becoming an asymptomatic carrier, while in the presence of impaired T-cell immunity, parasite replication increases, resulting in overt disease. In regions that are endemic for VL, most infected individuals remain asymptomatic [9] due to the host-parasite co-evolution; their role as ‘parasite carriers’ with capacity to infect sand flies is under careful consideration [10]. Furthermore, regardless of the causative *Leishmania* species, antileishmanial treatment does not provide a sterile cure; the parasite persisting in the host can cause VL relapses in case of immunosuppression [11,12].

An asymptomatically infected individual is defined as someone from an endemic area who shows an immune response (either antibodies or a specific cellular response) against *Leishmania*, or who has parasites or parasitic DNA in the blood, but remains healthy [13,14,15]; therefore, serological and molecular tests as well as tests detecting specific cell-mediated immunity have been interchangeably employed in numerous studies to identify latent leishmaniasis. No consensus has been established on tests for identification of latent leishmaniasis as existing diagnostic methods are not entirely suitable for detection of asymptomatic carriers [16].

A number of studies have used serological tests to identify this asymptomatic infection; *Leishmania*-specific antibodies have been detected by rk39 immunochromatographic tests, immunoenzymatic tests, direct agglutination tests and immunofluorescent antibody titration [17,18,19]; nevertheless these tests may exhibit scarce sensitivity in identification of latent leishmaniasis [20].

Direct diagnostic methods such as microscopy of splenic aspirates are unethical in asymptomatic individuals and are unsuitable for the surveillance of a large population [18], while molecular methods, such as PCR, exhibit higher sensitivity than microscopic examination and could detect parasitic DNA in peripheral blood [21]. However, while parasitemia is usually high in patients with VL, parasite levels can be low or undetectable in peripheral blood of asymptomatic hosts, showing fluctuations due to the short half-life of the *Leishmania* DNA [8,22,23]. For this reason, it has been suggested that PCR should not be used alone to identify asymptomatically infected individuals [8].

Anti-leishmanial cell-mediated immunity can also be assessed; the leishmanin skin test (LST) evaluates the degree of responsiveness to parasites by measuring the delayed-type hypersensitivity immune response to intradermal injection of *Leishmania* antigens [4]. However, the employment of LST is limited by the unviability of good manufacturing practice-grade reagents. Recently, the Whole Blood stimulating Assay (WBA)–a test for *Leishmani*a-specific cell immunity–has been developed to detect asymptomatically infected individuals. This assay is based on *ex vivo* stimulation of peripheral blood mononuclear cells with soluble *Leishmania* antigen (SLA), subsequently assessing the release of chemokines and cytokines by activated specific T lymphocytes [24].

Since the results obtained by employing a single test to identify latent leishmaniasis are highly variable, the combination of serological, cellular and molecular approaches has been pointed out to accurately detect asymptomatic infections [8]. In this work, we aimed to evaluate the prevalence of asymptomatic *Leishmania* infection in a *L*. *infantum* endemic area of northeastern Italy. For reaching this objective, we used a combination of methods, including molecular, serological, and cellular tests to detect latent leishmaniasis in individuals living in the examined area.

## Methods

### Ethics statement

The study was approved by the Ethics Committee of the Area Vasta Emilia Centro (CE-AVEC) with protocol number 764/2019. All participants provided their written informed consent, and all analyzed data were anonymized.

### Study cohort

Between October 2019 and August 2020, the Department of Public Health of the Bologna Local Health Unit (LHU) in northeastern Italy enrolled a cohort of 145 adult volunteers residing in a selected area of the Pianoro municipality (Bologna province), with the aim to perform a screening for asymptomatic *Leishmania* infection. An area of 2.3 km^2^ within the municipality of Pianoro was selected; in this small area, corresponding to about the 0.06% of the territory of the LHU of Bologna, originated 12% (i.e 11 out of 90 cases) of the autochthonous cases of human leishmaniasis that were notified to the LHU of Bologna between 2004 and 2018 (Todeschini R, personal communication). In the entire municipality of Pianoro (107.13 km^2^), no autochthonous cases of human leishmaniasis were notified in the previous decade (Todeschini R, personal communication), thus the Pianoro municipality is considered a recent focus of human leishmaniasis in northeastern Italy.

As inclusion criteria, adult individuals residing in the selected municipality for at least 5 years and with no history of cutaneous or visceral leishmaniasis were recruited. A sample of peripheral blood was collected from each participant after signing informed consent. Age, sex, country of birth, time of residence in Pianoro were recorded. For each individual, 10 ml of peripheral blood were collected and divided into 3 test tubes; one tube with ethylenediamine tetraacetic acid (EDTA) as anticoagulant, one tube with heparin as anticoagulant, and one tube without anticoagulant. Samples were transported to the Microbiology Unit at the University Hospital of Bologna (Italy) within 24 hours after collection.

We defined as asymptomatic *Leishmania* infection each case that tested positive to at least one of the screening tests, i.e Real-Time PCR, Western Blot (WB), and/or WBA.

### DNA extraction and Real-Time PCR

*Leishmania* DNA was extracted from 200 μl of EDTA peripheral blood by a semi-automatic DNA extraction system, using PROMEGA Maxwell 16 LEV Blood DNA kit on the Maxwell 16 instrument for the detection of parasitic DNA in the blood as described [16]. Briefly, PCR reactions were performed amplifying the kinetoplast DNA (kDNA) of *Leishmania*, used as a target for quantitative Real-Time PCR [23]. Primers (15 pmol of RV1 5’-CTTTTCTGGTCCTCCGGGTAGG-3’,15 pmol of RV2 5’-CCACCCGGCCCTATTTTACACCAA-3’) were synthesized by Integrated DNA Technologies—IDT (Leuven, Belgium), and 50 pmol of TaqMan probe (FAM-TTTTCGCAGAACGCCCCTACCCGC-TAMRA) were synthesized by Takara Bio (Mountain View, California); β2-microglobulin Real-Time PCR assay was run simultaneously as control of amplification. PCR was performed using 5 μl of DNA in 25 μl total reaction volume in 1x PREMIX Ex Taq Perfect Real Time (Takara Bio). PCR was considered positive for *Leishmania* DNA when an amplification curve at threshold cycle (Ct) < 40 was detected. Parasite quantification was accomplished by means of a standard curve which consisted of serial dilutions of *L*. *infantum* DNA (strain MHOM/TN/80/IPT1) ranging from the equivalent of 5*10^6^ to 5*10^−2^ parasites/ml. The parasitic load was expressed as equivalent parasites/ml of blood, since multiple copies of the molecular target are present in each protozoan cell [23].

### Western blot

The detection of specific *Leishmania* IgG in serum of volunteers was assessed employing the *Leishmania* WESTERN BLOT IgG kit (LDBio Diagnostics, Lyon, France), which identifies IgG against two leishmanial antigens, i.e 14-kDa and 16-kDa proteins. The positivity of the p14 and/or p16 bands is considered a sensitive and specific evidence of anti-*Leishmania* IgG in the tested serum [25].

### Whole blood assay

WBA was assessed to study the cytokine release by leukocytes upon stimulation with *Leishmania* antigens (JPC strain, MCAN/ES/98/LLM-722) as previously described [24]. IL-2 was quantified in 50 μl of plasma previously stimulated for 24h in tubes containing SLA prepared from stationary phase promastigote cultures [13,26]. The quantification of IL-2 was performed using Cytometric Bead Array Human Soluble Protein Flex Set (Becton Dickinson, Franklin Lakes, NJ, USA) and the method was performed using the BD FACSCanto cytofluorometer (Becton Dickinson, Franklin Lakes, NJ, USA). Results for IL-2 were reported as the difference between SLA-stimulated and control plasma concentrations as described in [24] and expressed in pg/ml. The cut-off (46.86 pg/ml) was determined by calculating the area under the receiver operating characteristic curve (AUC) and the 95% confidence intervals (CI) [0.8877 (0.7967–0.9787)] (S1 Fig). Although scanty unspecific reactions have been described with SLA stimulation in patients with tuberculosis, brucellosis, typhoid fever, malaria or trypanosomiasis, it is unlikely that the included subjects had any of these infections, as pathogens causing the abovementioned diseases are scarcely or not circulating in northern Italy.

### Statistical analysis

Statistical analyses were performed with the use of descriptive statistics including mean, median value, minimum and maximum, and percentages. To determine if data were distributed normally, tests of equal proportions among all variables were performed employing the χ^2^ test. Differences were considered significant only when the p value was below 0.05. The cut-off value for IL-2 was determined by calculating the AUC plus the 95% CI. To measure the relationship between WB and WBA, and between PCR and WB or PCR and WBA, Pearson’s correlation coefficients were calculated using GraphPad Prism 8.0 (GraphPad Software). To measure the concordance between different methods, the Kappa value was calculated. Results were interpreted according to the following Kappa values: i) 0.01–0.20, slight agreement; ii) 0.21–0.40, fair agreement; iii) 0.41–0.60, moderate agreement; iv) 0.61–0.80, substantial agreement; and v) 0.81–1.00, perfect agreement.

The numerical data used in the Figures and Tables are held in a public repository (https://doi.org/10.4321/repisalud.14600).

## Results

Our study cohort included 145 adult volunteers, with a mean age of 58 years. All individuals were of Caucasian ethnicity; 143 volunteers were born in Italy, while 2 individuals were born in a different European country (Germany and Spain, respectively). Eighty-five % of the recruited individuals (124/145) were living in the Pianoro municipality for more than 10 years.

Based on the above-mentioned definition of asymptomatic *Leishmania* infection, we considered as positive each individual that tested positive to at least one of the three techniques. Twenty-three out of 145 volunteers were positive, indicating a prevalence of 15.9%; 15 out of 145 subjects tested positive to WB (10.3%, Table 1). In detail, 4 subjects showed positivity to the p14 band (26.5%), 7 to the p16 band (47.0%), and 4 to both p14 and p16 bands (26.5%).

**Table 1:** Rates of asymptomatic *Leishmania* infection among volunteers residing in Pianoro, Bologna Province (northeastern Italy), as evaluated by Western Blot, Real-Time PCR and WBA.

**No. of subjects (145)**	**WB Pos** **No. (%)**	**PCR Pos** **No. (%)**	**WBA Pos** **No. (%)**
	15 (10.3%)	6 (4.1%)	12 (8.3%)

No; number, WB; Western Blot, PCR; Polymerase Chain Reaction, WBA; Whole blood assay, Pos; Positive.

Molecular screening was performed to detect the presence of kDNA of *Leishmania* in peripheral blood using a Real-Time PCR. Among the 145 volunteers included in the study, 6 individuals carried leishmanial DNA in the peripheral blood (4.1%, Table 1); the parasitemia was low, with a median parasite load of 8.24 x 10^−1^ /ml of blood (interquartile range, IQR, 3.79 x 10^−2^–2.39 x 10^−1^).

The WBA was used to evaluate the specific cell-mediated response of the volunteers after blood stimulation with *Leishmania*-specific antigens. Of the 145 subjects, 12 tested positive (8.3%, Table 1); these individuals exhibited an IL-2 value above the cut-off of positivity with a median IL-2 concentration of 213 pg/ml (IQR, 142–878).

As shown in Table 2, we did not observe any difference in terms of positivity for *Leishmania* infection by segregating the volunteers according to sex and age.

**Table 2:** Demographic characteristics of 145 volunteers residing in Pianoro municipality, Bologna Province (northeastern Italy).

Variables (No. of individuals)	WB pos No. (%)	PCR pos No. (%)	WBA pos No. (%)	p-value*
**Sex**				
Male **(67)**	7 (10.4%)	1 (1.5%)	6 (8.9%)	0.23
Female **(78)**	8 (10.2%)	5 (6.4%)	6 (7.7%)	
**Age (years)**				
19–42 **(26)**	4 (15.4%)	3 (11.5%)	3 (11.5%)	0.11
43–66 **(70)**	4 (5.7%)	2 (2.9%)	4 (5.7%)	
67–90 **(49)**	7 (14.3%)	1 (2.0%)	5 (10.2%)	

No.; number of individuals, WB; Western Blot, PCR; Polymerase Chain Reaction, WBA; Whole blood assay, Pos; Positive *; χ^2^ test was employed.

**Fig 1:**
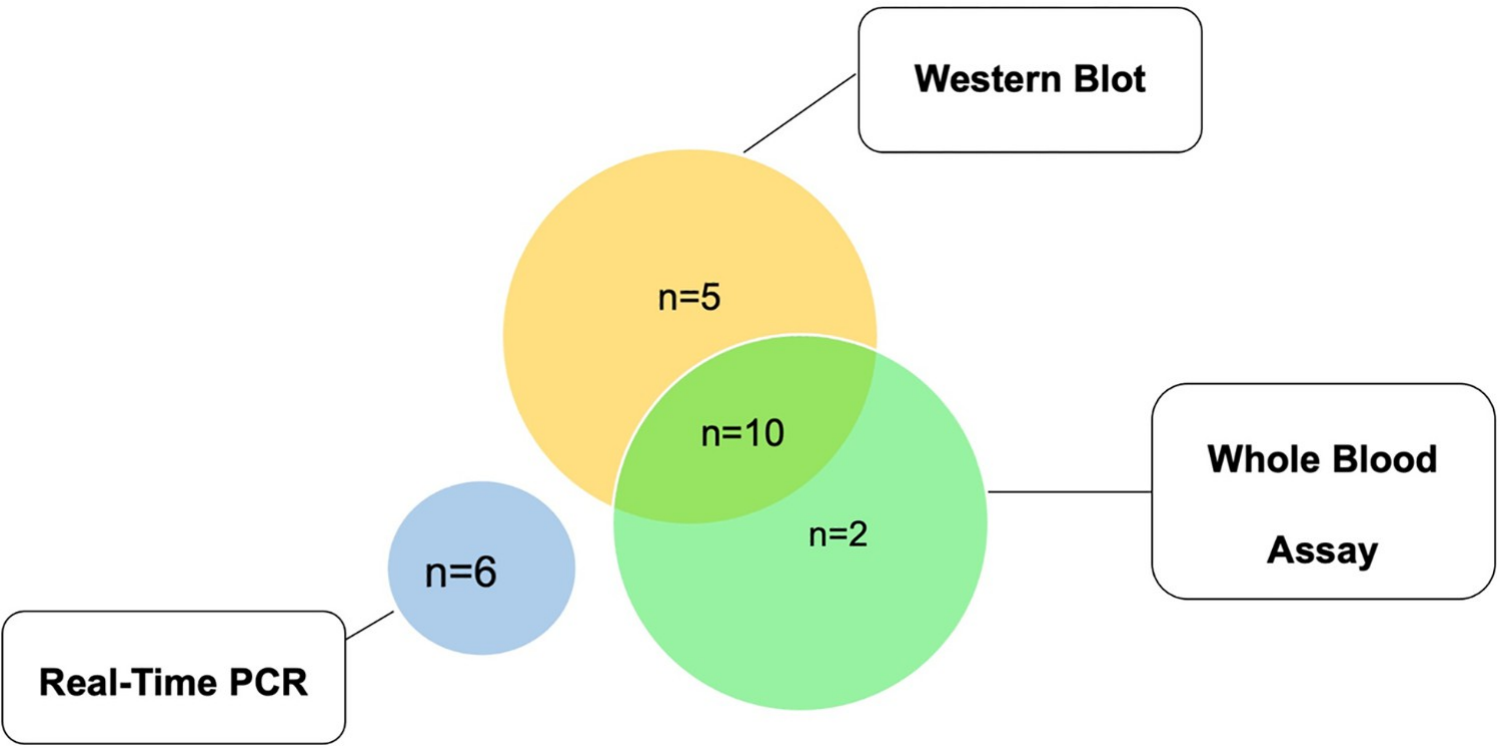
Type of positive *Leishmania* markers, n = 23 individuals that tested positive to at least one of the three methods.

If we analyze the prevalence combining the screening tests, the overall prevalence of *Leishmania* infection was 15.9%. We identified 6 patients positive for kDNA PCR in blood. None of the subjects that tested positive for kDNA were also positive for serology or specific cell-mediated immunity. When comparing the immunological methods, we observed that 10 individuals tested positive for both WB and WBA, while 5 and 2 subjects were positive only for WB or WBA, respectively (Fig 1). These two techniques (WB and WBA) showed a quite strong correlation (Pearson’s r = 0.6980; 95% CI: 0.59–0.78), while the Kappa value was 0.63 (63%), which indicates a rate of substantial concordance between WB and WBA.

We examined the results of tests for the identification of *Leishmania* infection by considering the timing of blood collection (S1 Table). Most of the blood samples were taken in the autumn-winter months (93% of the total), while only 7% of specimens were collected between June and August. Interestingly, considering the individuals testing positive for parasitic DNA (n = 6), they were all enrolled in the study in November 2019. Conversely, positivity to specific antibodies and cell-mediated immunity was detected in samples that were collected in different months between October 2019 and August 2020.

## Discussion

Combining different tests substantially increased the yield of positivity in detecting latent *Leishmania* infection. Among the 145 individuals that were living in a municipality of the Bologna province (northeastern Italy) and that were recruited and screened for *Leishmania* infection, 23 (15.9%) subjects tested positive to one or more tests. We observed that all age groups were similarly affected by latent leishmaniasis; this suggests that the residents of the Pianoro municipality were exposed to the *Leishmania* parasite in recent years, so that individuals belonging to all age groups that were infected developed immunity in the same time interval. In addition, no significant difference was observed by sex in the prevalence rate of asymptomatic *Leishmania* infection, which is in line with our previous observations in southern Europe [16,17]. More generally, although there is a higher frequency of VL in males than female [27], most of the studies published about asymptomatic *Leishmania* infection reported no differences regarding sex [28].

By employing the test combination of Real-Time PCR, WB, and WBA, we found that the prevalence of asymptomatic *Leishmania* infection in individuals residing in the Pianoro municipality was 15.9%. This percentage is similar to what we observed in a previous study in 240 blood donors living in another municipality of the Bologna province at high risk for leishmaniasis, i.e. Valsamoggia (about 19 km from Pianoro). In the mentioned study, in fact, a prevalence of 12.5% was displayed in blood donors by employing a combination of WB and kDNA PCR [16]. Apart from the obvious differences between the studies (including year and type of study design, type of cohort, study area, etc), it is likely that adding a test for *Leishmania*-specific cell-mediated immunity (WBA) in the current study has allowed the detection of asymptomatic carriers that would have not been discovered otherwise.

In the current study, among 23 *Leishmania* positive individuals, 10 volunteers tested positive for both WBA and WB, showing a quite strong correlation and a substantial agreement between the two immunological methods, even if they detect different arms of the anti-leishmanial immune response. Conversely, there was no correlation and no agreement comparing the results of kDNA-PCR with the immunological tests as none of the subjects that tested positive for kDNA in the blood was also positive for the specific cell-mediated response and/or for the presence of *Leishmania* antibodies in sera. The observation of discordant results between molecular methods and serological methods in detection of asymptomatic *Leishmania* infection is in line with previous studies performed in the Mediterranean Europe and India [16,24,29].

The presence of parasites in the blood is transient in asymptomatic subjects, which is why the parasites are not frequently detected by PCR [8]. In our study, all individuals that tested positive for parasitic DNA in the blood were enrolled in November 2019 (S1 Table). An association between the presence of parasitic DNA in the blood and recent exposure during the sand fly transmission period (May–August 2019) is possible, but our study group is too small to draw any conclusion on this matter.

The ability of T cells to react against *Leishmania* contributes to the maintenance of an asymptomatic state of the infected host; in the absence of immunosuppression a positive test measuring specific cell-mediated immunity indicates a low risk to develop VL [30]. Thus, specific T cells against *Leishmania* would keep parasite replication under control, supporting our abovementioned finding that PCR-positive individuals did not exhibit a positive WBA. This observation is also linked to the evidence that most asymptomatic subjects with strong anti-*Leishmania* cell-mediated response and/or positive serology are not able to infect sandflies in xenodiagnosis studies [31,32].

To date, a single screening test for asymptomatic *Leishmania* infection has not been identified and conventional diagnostic methods are insufficient in detecting latent infection. The strength of this study was to select three sensitive assays to detect *Leishmania* infection (kDNA Real-Time PCR, WB and WBA) and ensuring that a case with a positive result of any test within the combination was counted as positive. By selecting the most sensitive methods that are available for the diagnosis of VL [20,21,25,33] and for the detection of anti-leishmanial cell mediated response [11,24], we believe that the sensitivity of our test combination in detecting latent leishmaniasis is high.

The lack of a gold standard for the identification of asymptomatic *Leishmania* infection is the main limitation of the study as there is no means to calculate the specificity of the selected tests. It also remains to be defined whether serological tests and tests evaluating the specific T-cell immunity can identify asymptomatic carriers of *Leishmania* or if they just indicate past exposure to the parasite. In addition, the study was conducted on a limited number of participants, and other studies should be performed to validate the use of the suggested methods to detect latent leishmaniasis.

Finally, we cannot exclude that potentially cross-reactive infectious agents circulate in the area where the study was performed. Recently, residents in areas from Central Italy and from the Pelagie islands (southern Italy) were found to be exposed to or infected with *Leishmania tarentolae*, which has been historically considered a saurian-associated *Leishmania* species [34,35]. So far, no studies have identified *L*.*tarentolae* in northern Italy, but further studies would be needed to rule out a cross-reactivity between *L*.*infantum* and *L*.*tarentolae* in our sample‘s cohort.

Limited information is available regarding the role of individuals with asymptomatic infection in the transmission of *L*.*infantum*, but recent evidence suggests that asymptomatic carriers can be reservoirs of parasites during *Leishmania* life cycle and contribute to transmission [31,36]. Moreover, this protozoan infection can reactivate if asymptomatic carriers are immunocompromised, with the development of full-blown VL [11,12]. Thus, establishing the tools to identify *Leishmania* carriers is of importance not only for screening individuals at risk of overt disease, but also for elimination/control programmes. In conclusion, the prevalence of latent *L*.*infantum* infection in an endemic area in northeastern Italy was 15.9%. Combining different tests substantially increased the yield of positivity in detecting latent *Leishmania* infection. The test combination including kDNA PCR and immunological tests appears to be effective to identify latent leishmaniasis in an endemic area.

## Supporting information

S1 Fig Receiver operating characteristic (ROC) curve for the prediction of latent asymptomatic infected individuals in an endemic area of *L*. *infantum* (Italy), based on IL-2 levels in stimulated plasma. (TIF)

S1 Table Results of tests for the identification of *Leishmania* infection by the timing of blood sampling. (DOCX)
