# Supplementary figures and images for: Test combination to detect latent Leishmania infection: A prevalence study in a newly endemic area for L. infantum, northeastern Italy
Source: PLoS Negl Trop Dis. 2022 Aug 15;16(8):e0010676. doi: 10.1371/journal.pntd.0010676 (PMC9410555; doi:10.1371/journal.pntd.0010676)

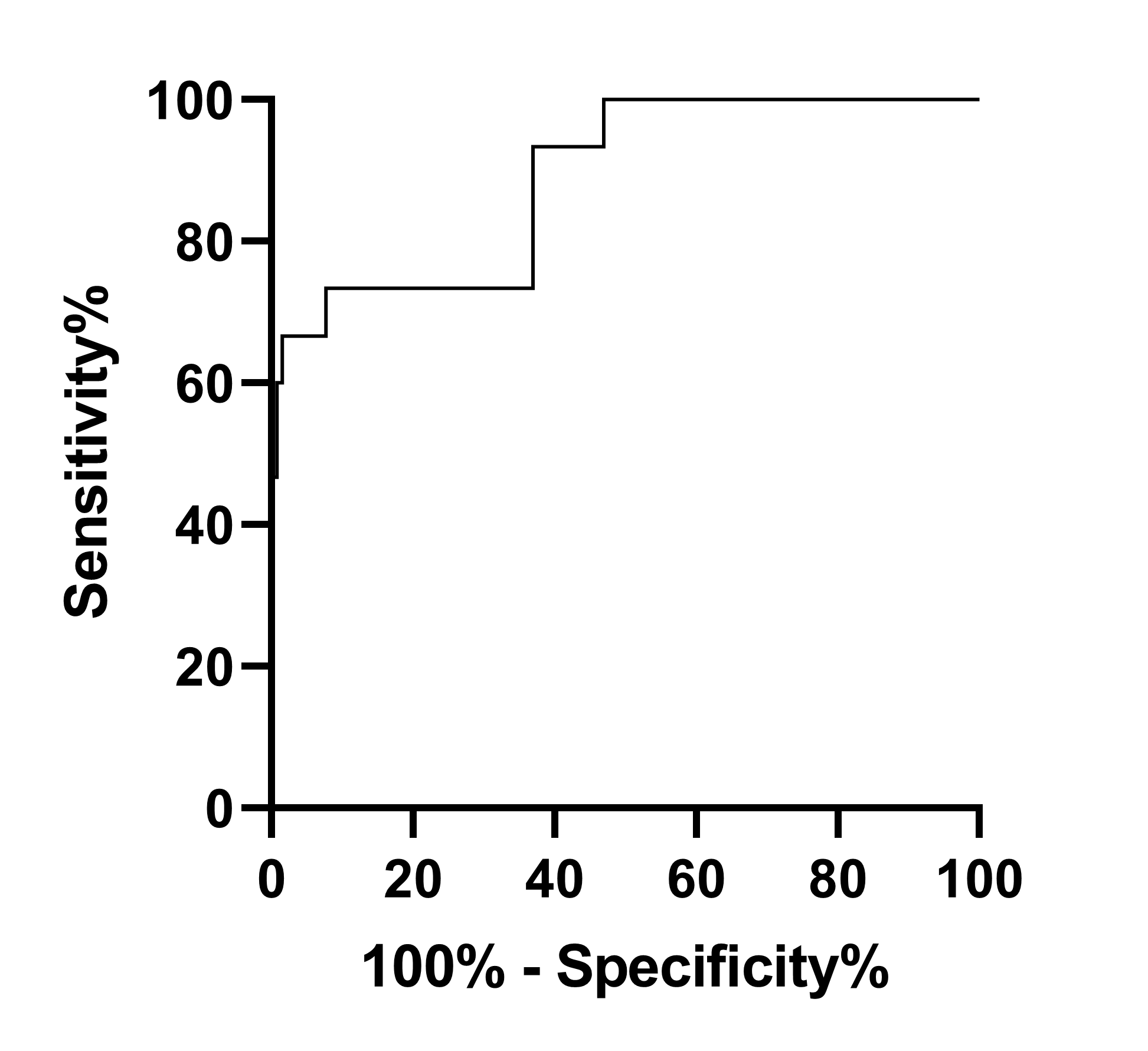

Supplement: S1 Fig — (TIF) [file pntd.0010676.s001.tif]
