# Supplementary material for: Test combination to detect latent Leishmania infection: A prevalence study in a newly endemic area for L. infantum, northeastern Italy
Source: PLoS Negl Trop Dis. 2022 Aug 15;16(8):e0010676. doi: 10.1371/journal.pntd.0010676 (PMC9410555; doi:10.1371/journal.pntd.0010676)
Supplement: S1 Table — (DOCX) [file pntd.0010676.s002.docx]

**Results of tests for the identification of *Leishmania* infection by the timing of blood sampling.**

| **Date of blood collection** | **Total blood samples (n= 145)** | **Positive result for *Leishmania* infection (number and %)** | **Positive result at PCR (number and %)** | **Positive result at WBA and/or WB (number and %)** |
| --- | --- | --- | --- | --- |
| October 2019 | 32 | 3 (13.1%) | 0 (0%) | 3 (17.7%) |
| November 2019 | 46 | 9 (39.1%) | 6 (100%) | 3 (17.6%) |
| February 2020 | 57 | 8 (34.8%) | 0 (0%) | 8 (47.1%) |
| June-July 2020 | 5 | 1 (4.3%) | 0 (0%) | 1 (5.9%) |
| August 2020 | 5 | 2 (8.7%) | 0 (0%) | 2 (11.8%) |

WBA; whole blood assay, WB; Western Blot
